# Premature ventricular contraction arising from the left coronary sinus cusp: Which signal is the target of ablation?

**DOI:** 10.1016/j.ipej.2024.09.003

**Published:** 2024-09-10

**Authors:** Takashi Nakashima, Masaru Nagase, Shigekiyo Takahashi, Takuma Aoyama

**Affiliations:** a Department of Cardiology, Central Japan International Medical Center, Gifu, Japan; b Department of Cardiology, Graduate School of Medicine, Gifu University, Gifu, Japan; c Department of Molecular Pathophysiology, Shinshu University Graduate School of Medicine, Matsumoto, Japan

**Keywords:** Artifact, Coronary sinus cusp, Premature ventricular contraction, Radiofrequency catheter ablation

## Abstract

We described a premature ventricular contraction arising from the left coronary sinus cusp, in which we discussed about the interpretations of the signals recorded there. Our case provided further insights into the interpretation of signals recorded at the coronary sinus cusp during premature ventricular contraction ablation.

## 1 Case

A 69-year-old woman was referred for radiofrequency catheter ablation (RFCA) of symptomatic premature ventricular contractions (PVC). No significant coronary stenosis was observed on computed tomography angiography. Echocardiography revealed no evidence of structural heart disease and an ejection fraction of 60 %.

**Fig. 1:**
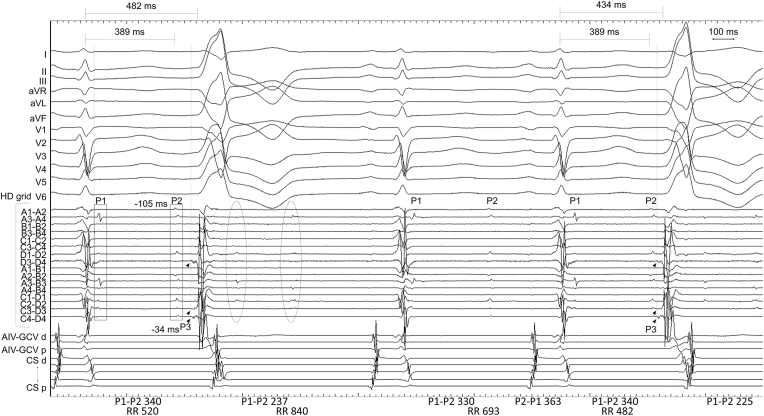
Intracardiac electrogram recorded using a grid-mapping catheter positioned at the left coronary sinus cusp (LCC). P1 and P2 amplitudes and their durations range from 0.11 to 0.26 mV and 21–33 ms, respectively. Vertical lines represent the QRS offset and PVC onset. Numbers are shown in milliseconds. AIV-GCV, anterior interventricular vein-great cardiac vein; CS, coronary sinus; P1-P2, P1-P2 interval; P2-P1, P2-P1 interval; RR, RR interval; d, distal; p, proximal. Please refer to the text for further discussion.

At baseline, the PVC exhibited a left bundle branch block morphology, an inferior axis, and a precordial R-wave transition in V3 (PVC1). Mapping of the right ventricular outflow tract with a three-dimensional mapping system (EnSite; Abbott, St. Paul, MN, USA) and a high-density grid-mapping catheter (Advisor HD Grid Mapping Catheter; Abbott St. Paul, MN, USA) revealed a prepotential preceding QRS onset by 27 ms at the septum. RFCA performed at this site suppressed PVC1. However, provocative isoproterenol induced PVC with a slight change in QRS morphology (PVC2; Fig. 1). Subsequently, mapping was performed at the left ventricular outflow tract, coronary sinus cusp, and distal coronary sinus. Intracardiac electrograms recorded using a grid-mapping catheter positioned at the left coronary sinus cusp (LCC) are shown in Fig. 1 (a sweep speed 100 mm/s). How can these signals be interpreted? Which signal is the target of ablation?

## 2 Discussion

**Fig. 2:**
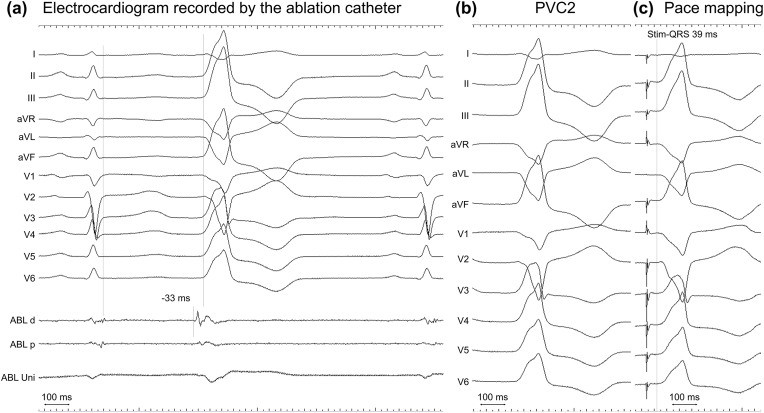
Intracardiac electrogram recorded by the ablation catheter and pace mapping. **(A)** The ablation catheter is positioned at the LCC. The electrograms corresponding to P1 and P2 are not evident. The vertical lines represent sinus-QRS offset and PVC onset. **(B)** A surface-twelve lead electrocardiogram of PVC2. **(C)** Pace mapping performed at the LCC using an ablation catheter. The vertical lines represent the onset of PVC.

One signal (P1; square) was recorded immediately after the local ventricular signal during the sinus rhythm. The other signal (P2; square) was recorded at the terminal portion of the T-wave and preceded the PVC-QRS onset by 105 ms. The third signal (P3; arrowheads) was recorded and preceded PVC-QRS onset by 34 ms. Other signals appeared after PVC2 (two circles). During sinus rhythm (the 3rd and 4th QRS complexes in Fig. 1), the sum of intervals P1-P2 (330 ms) and P2-P1 (363 ms) was equal to the RR interval (693 ms). Although the P1-P2 interval before PVC (340 ms) and the coupling interval between the sinus-QRS complex and P2 (389 ms) were fixed, the interval between the sinus-QRS complex and P3 changed; consequently, the coupling interval between the preceding sinus-QRS complex and PVC2 changed (482 ms–434 ms). Pacing from the grid-mapping catheter failed to capture the myocardium. The ablation catheter positioned at the LCC recorded a prepotential preceding the PVC onset by 33 ms (Fig. 2A). Ablation catheter did not record the P1 and P2 signals recorded by the grid-mapping catheter. The catheter orientations to tissue, and the differences in electrode size or interelectrode distance were probably attributed to these results [1]. Pacing from the ablation catheter produced a paced-QRS morphology similar to that of PVC2, with a latency of 39 ms (Fig. 2B and C). These findings suggested that P1 and P2 signals could be aortic valve-related (opening and closing) artifacts [2,3], and that the pacing captured the P3, indicating the preferential pathway capture, and this site, that is, P3, could be the target to ablate [4,5]. Application of RFCA at this site successfully eliminated PVC2. Provocation of isoproterenol did not induce PVC. However, the P1 and P2 signals were still present.

Previous studies have reported that aortic valve-related (opening and closing) artifacts could mislead the interpretation of the target signals of ablation for PVC arising from the left-side outflow tract [2,3]. However, these studies did not provide intracardiac electrograms recording both the aortic valve-related artifact and the prepotential, which can be the appropriate target signal to ablate. Our case provided further insights into the interpretation of signals recorded at the coronary sinus cusp during PVC ablation.

## Conflicts of interest

The authors declare that they have no known competing financial interests or personal relationships that could have appeared to influence the work reported in this paper.

none

## Funding

none.

## Approval of the research protocol

This case report was approved by the Institutional Review Board of the Central Japan International Medical Center.

## Informed consent

The patient was provided informed consent in written form.

## Ethical statement

This case report was approved by the Institutional Review Board of the Central Japan International Medical Center. The patient was provided informed consent in written form.

## Declaration of competing interest

The authors declare that they have no known competing financial interests or personal relationships that could have appeared to influence the work reported in this paper.
